# Effects of Extracts from Thai Piperaceae Plants against Infection with *Toxoplasma gondii*

**DOI:** 10.1371/journal.pone.0156116

**Published:** 2016-05-23

**Authors:** Arpron Leesombun, Sookruetai Boonmasawai, Naomi Shimoda, Yoshifumi Nishikawa

**Affiliations:** 1 National Research Center for Protozoan Diseases, Obihiro University of Agriculture and Veterinary Medicine, Inada-cho, Obihiro, Hokkaido 080–8555, Japan; 2 Department of Preclinic and Applied Animal Science, Faculty of Veterinary Science, Mahidol University, 999 Phutthamonthon Sai 4 Road Salaya, Phutthamonthon Nakhonpathom 73170, Thailand; Ehime University, JAPAN

## Abstract

Herbal medicines and natural herb extracts are widely used as alternative treatments for various parasitic diseases, and such extracts may also have potential to decrease the side effects of the standard regimen drugs used to treat toxoplasmosis (sulfadiazine-pyrimethamine combination). We evaluated how effective the Thai piperaceae plants *Piper betle*, *P*. *nigrum* and *P*. *sarmentosum* are against *Toxoplasma gondii* infection *in vitro* and *in vivo*. Individually, we extracted the piperaceae plants with ethanol, passed them through a rotary evaporator and then lyophilized them to obtain crude extracts for each one. The *in vitro* study indicated that the *P*. *betle* extract was the most effective extract at inhibiting parasite growth in HFF cells (IC_50_ on RH-GFP: 23.2 μg/mL, IC_50_ on PLK-GFP: 21.4 μg/mL). Furthermore, treatment of experimental mice with the *P*. *betle* extract for 7 days after infection with 1,000 tachyzoites of the *T*. *gondii* PLK strain increased their survival (survival rates: 100% in 400 mg/kg-treated, 83.3% in 100 mg/kg-treated, 33.3% in 25 mg/kg-treated, 33.3% in untreated mice). Furthermore, treatment with 400 mg/kg of the *P*. *betle* extract resulted in 100% mouse survival following infection with 100,000 tachyzoites. The present study shows that *P*. *betle* extract has the potential to act as a medical plant for the treatment of toxoplasmosis.

## Introduction

*Toxoplasma gondii*, an obligate intracellular protozoan, causes toxoplasmosis. Infection with *T*. *gondii* threatens one-third of the global human population [1]. *Toxoplasma* infections have nonspecific symptoms, but can be associated with several clinical syndromes and cause serious complications and severe life-threatening disease in congenitally infected and immunocompromised hosts. Ingestion of raw or undercooked meat containing *T*. *gondii* tissue cysts is the main route of infection for this parasite [2,3]. Currently, sulfonamide drugs and pyrimethamine used in combination are the gold-standard medicines for treating toxoplasmosis [4]. These drugs have a synergistic activity against tachyzoites, but have limited efficacy in eliminating *T*. *gondii* encysted bradyzoites [5], and severe side effects and adverse drug reactions such as hematological reactions, embryopathies, bone marrow supression, hypersensitivity, and gastrointestinal disorders have been noted [6,7]. Therefore, the development of novel efficacious drugs with low toxicities is urgently needed.

Piperaceae plants comprise approximately 1,000 species of herbs, and are found in tropical areas of India, Southeast Asia and Africa [8]. Forty species of such plants have been identified in Thailand [9]. These plants are used as active ingredients in Thai traditional medicine and have many uses, such as ameliorating stress, improving digestion and nutrient absorption, and balancing general health; they also have antimalarial properties and are used for cancer treatment [10,11,12,13]. Pharmacologically, the properties of piperaceae plants have been shown to be antibacterial, antioxidant, gastro protective, and anticancer [14]. Furthermore, such plants have been shown to have anti-leishmanial activity [15] and anti-malarial activity [16,17]. Because herbal medicines and natural herb extracts are widely used as alternative treatments for various parasitic diseases and some have been tested on *T*. *gondii in vitro* [18], we were interested in exploring whether piperaceae plants possess anti-*Toxoplasma* activity. Thus, the aim of this study was to evaluate the effects of ethanol extracts from Thai piperaceae plants (*P*. *betle*, *P*. *nigrum* and *P*. *sarmentosum*) on *T*. *gondii* infections *in vitro* and *in vivo*. Our data indicate that of the three plants that we tested, *P*. *betle* extract has the potential to act as a medical plant for treating toxoplasmosis.

## Materials and Methods

### Ethics statement

This study was performed in strict accordance with the recommendations in the Guide for the Care and Use of Laboratory Animals of the Ministry of Education, Culture, Sports, Science and Technology, Japan. The protocol was approved by the Committee on the Ethics of Animal Experiments of the Obihiro University of Agriculture and Veterinary Medicine (Permit number 27–30, 28–46). All surgery was performed under isoflurane anesthesia, and all efforts were made to minimize animal suffering.

### Animals

Experiments were performed using female C57BL/6 mice (6–8 weeks old) obtained from Clea Japan, Inc. Six mice per cage were kept in the animal facility at the National Research Center for Protozoan Diseases (Obihiro University of Agriculture and Veterinary Medicine, Obihiro, Japan) under standard laboratory conditions with commercial food and water available *ad libitum*.

### Parasites and cell cultures

The two strains of *T*. *gondii* used for the experiments, RH-GFP (a green fluorescent protein expressing-RH strain) [19] and PLK-GFP [20] (a PLK strain), were maintained in African green monkey kidney (Vero) cells cultured in minimum essential medium eagle (MEM, Sigma, St. Louis, MO, USA) supplemented with 8% heat-inactivated fetal bovine serum (FBS) and 100 U/mL penicillin, 10 mg/mL streptomycin at 37°C in a 5% CO_2_ atmosphere. Parasites were propagated every three days. Parasites were purified by washing them in cold phosphate-buffered saline (PBS), and the final pellet was resuspended in cold PBS and passed three times through a 27-gauge needle syringe. Next, the parasites were filtered through a 5.0 μm pore filter (Millipore, Bedford, MA), washed twice with 10 mL of PBS, and centrifuged at 1,000 × *g* for 10 min. The parasites were refiltered and their numbers counted on a hemacytometer for each experiment. Human foreskin fibroblast (HFF) cells were maintained in Dulbecco's modified Eagle’s medium (DMEM, Sigma) supplemented with 10% FBS, 100 U/mL penicillin, and 10 mg/mL streptomycin at 37°C in a 5% CO_2_ atmosphere.

### Plant materials

Three types of piperaceae plants were used in this study. The fresh leaves of *Piper betle* L. 8,955 g and *P*. *sarmentosum* Roxb. 11,310 g were purchased from Don Wai Floating market in Nakhon Pathom province, and the 1,000 g dried seeds of *Piper nigrum* L., were purchased from herbal drug stores (Vejpong Pharmacy Co., Ltd) Thailand. All plant materials were identified by the Faculty of Pharmacy, Mahidol University. The plant serial numbers for *P*. *betle* L. are as follows: PBM05160, *P*. *nigrum* L.: PBM05159, *P*. *sarmentosum* Roxb.: PBM05161 (S1 Fig). The fresh leaves were cleaned with water. Only sound leaves were dried in a hot air oven at 70°C for 48 h, after which they were ground into small pieces. The extraction methods were modified from Choochote et al [21]. Plant materials were extracted once with 97% ethanol (Sigma-Aldrich, St. Louis, MO, USA) at room temperature (RT) over a 3-day period. The solution was filtrated through sterile gauze and cotton, the filtrate was evaporated to dryness under reduced pressure at 40°C with a rotary evaporator (Rotavapor R-200/205, BÜCHI, Flawil, Switzerland), and then lyophilized using a Freeze Dry Vacuum System (Labconco, Kansas City, MO USA). The weights of the final crude extracts of *P*. *betle*, *P*. *sarmentosum* and *P*. *nigrum* were 43.42 g, 39.07 g and 51.18 g, respectively, and the yields of the extracts based on their dry weights [22] were 3.05%, 3.97% and 3.93%, respectively. The final crude extracts were dissolved in dimethyl sulfoxide (DMSO) at 100 mg/mL and kept at −30°C.

### Cytotoxicity tests

Cytotoxicity analysis of the three piperaceae crude ethanol plant extracts (*P*. *betle*, *P*. *nigrum*, *P*. *sarmentosum*) and sulfadiazine (Sigma-Aldrich) were conducted on HFF cells. Sulfadiazine was dissolved in 1-M NaOH (stock solution 200 mg/mL) according to the manufacturer’s recommendations. Because 0.01 M NaOH did not inhibit HFF cell growth, we used 1 mg/mL sulfadiazine at the highest concentration in our study. HFF cells were plated at 100 μl/well in 96-well plates (cell suspensions 1 × 10^5^ cells/mL in DMEM supplemented with 10% FBS), and then incubated at 37°C in a 5% CO_2_ atmosphere for 48 h. Next, the cells were exposed to the piperaceae extracts at final concentrations of 1, 5, 10, 25, 50, 100 μg/mL, sulfadiazine (at 10 ng/mL to 1 mg/mL), and culture medium was used as a control. After 24 h, the cell viability was measured by adding cell counting kit-8 (CCK-8, Dojindo Molecular Technologies, Inc. Japan) to the cultures. The absorbance of the supernatant was measured at 450 nm using an MTP-120 micro plate reader (Corona Electric, Ibaraki, Japan). HFF cell viability (%) is expressed as [(the absorbance of cells treated with the extracts / (the absorbance of cells cultured with medium alone) × 100].

### Indirect fluorescent antibody test (IFAT)

Vero cells, plated at 1 mL/well in 12-well plates (cell suspensions 1 × 10^5^ cells/mL in MEM supplemented with 8% FBS), were incubated at 37°C in a 5% CO_2_ atmosphere for 24 h. Coverslips were collected at 24 h after parasite inoculation, washed twice with PBS containing 1 mM CaCl_2_ and 1 mM MgCl_2_ (PBS++), and then fixed with 3% paraformaldehyde in PBS++. After washing twice with PBS++, the cells were permeabilized with 0.3% Triton X-100 in PBS++ for 5 min at RT. After washing, the coverslips were incubated with 3% bovine serum albumin (BSA) in PBS++ at RT for 30 min. To count the number of parasites in parasitophorous vacuoles (PVs), the coverslips were incubated with an anti-SAG1 monoclonal antibody (clone TP3; Advanced ImmunoChemical Inc., Long Beach, CA, USA) diluted 1:100 in 3% BSA in PBS++ for 1 h at RT. After washing three times with PBS++, the coverslips were incubated with Alexa Fluor 594-conjugated goat anti-mouse IgG (Sigma) diluted 1:1,000 in 3% BSA in PBS++ for 1 h at RT, and then washed again with PBS++. Nuclear DNA was labelled with Hoechst 33342 (1:10,000 dilution, Thermo Fisher Scientific Inc., MA, USA) for 30 min. The coverslips were placed on a glass slide coated with Mowiol (Calbiochem, San Diego, CA, USA), and the slides were examined using an All-in-one Fluorescence Microscope (BZ-9000, Keyence, Tokyo, Japan).

### Effects of piperaceae extracts on intracellular *T*. *gondii in vitro*

HFF cells, plated at 100 μl/well in 96-well plates (cell suspensions 1 × 10^5^ cells/mL in DMEM supplemented with 10% FBS), were incubated at 37°C in a 5% CO_2_ atmosphere for 48 h. To examine the effects of this treatment on the intracellular parasites, RH-GFP and PLK-GFP (5 × 10^4^ tachyzoites per well) were added to the wells for 4 h and the extracellular parasites were washed away. Then, the piperaceae extracts at final concentrations of 1, 5, 10, 25, 50, and 100 μg/mL (100 μl/well of media) were added for 72 h. Sulfadiazine (1 mg/mL, Sigma) and control media were used as positive and negative controls, respectively. The fluorescence intensity of RH-GFP and PLK-GFP were measured using a microplate reader (SH-900, Corona Electric Co., Ltd, Ibaraki, Japan). The correlation coefficient between the fluorescence intensity of GFP and the number of parasites (a two-fold serial dilution ranging from 1,000,000 to 7812.5 parasites) was calculated using the Pearson correlation coefficient and a positive correlation was confirmed (RH-GFP: r = 0.992, PLK-GFP: r = 0.969). The growth inhibition of RH-GFP and PLK-GFP (%) was expressed as follows: [(average fluorescence intensity of GFP with medium alone) − (the fluorescence intensity of GFP treated with either of the extracts or sulfadiazne) / (average fluorescence intensity of GFP with medium alone)] × 100. The half maximal inhibitory concentration (IC_50_) values of the plant extracts and sulfadiazine on *T*. *gondii* were calculated based on three independent experiments performed together by GraphPad Prism 5 software (GraphPad Software Inc., La Jolla, CA). Additionally, to measure *T*. *gondii* replication in vero cells, the PV sizes for RH-GFP expressed as a percentage (%) were determined by counting the number of parasites per PV (a total of 25 randomly selected vacuoles) at 24 h after infection (post-treatment with the extract as described above) based on the SAG1 signal measured by IFAT, as described above.

### Effects of piperaceae extracts on extracellular *T*. *gondii in vitro*

RH-GFP tachyzoites (2 × 10^5^) were pretreated with either of the three piperaceae extracts (25 μg/mL), sulfadiazine (1 mg/mL), or MEM alone (1 mL/tube) for 1 h at 37°C. Then, the pretreated parasites were added to vero cells at 1 mL/well in a 12-well plate (parasites per host cell ratio = 2:1). At 2–3 h post-infection, the extracellular parasites were washed away and MEM supplemented with 8% FBS was added. To determine the percentage inhibition of the *T*. *gondii* tachyzoites, the infection rates at 24 h post-infection were calculated by IFAT as follows: [(number of SAG1-positive vero cells) / (100 randomly selected vero cells)] × 100. The percentage inhibition of the tachyzoites was expressed as follows: [(average infection rate after treatment with medium alone) − (infection rate after treatment with either of the extracts) / (average infection rate after treatment with the medium alone)] × 100.

### Effects of the piperaceae extracts on *T*. *gondii* infections *in vivo*

Mice were intraperitoneally inoculated with *T*. *gondii* (PLK strain, 1 × 10^3^ tachyzoites/mouse). At 24 h post-infection, the mice were intraperitoneally injected with the *P*. *betle* extract at either 25 (n = 6), 100 (n = 12), and 400 mg/kg/24 h (n = 12), or PBS (n = 12) for 7 days. To further evaluate the anti-*Toxoplasma* activity of the *P*. *betle* extract, the mice infected with 1,000 and 100,000 PLK strain *T*. *gondii* tachyzoites (n = 6 per group) were treated with 400 mg/kg of *P*. *betle* extract or PBS via the intraperitoneal route, or 400 mg/L sulfadiazine via the drinking water as a standard treatment [23] for 7 days. The mice were observed daily for 30 days post-infection. Daily observations such as body weight, morbidity, mortality and clinical signs were noted, as were the clinical scores. Most of the signs recorded were assessed by the criteria used in other infection studies with protozoan parasites [24,25]. The scores varied from 0 (no signs) to 10 (all signs). The clinical signs recorded included hunching, piloerection, worm-seeking behavior, ptosis, sunken eyes, ataxia, latency of movement, deficient evacuation and touch reflexes, and lying on belly. The brains from the surviving mice were collected to determine the parasite burden by quantitative PCR, as described previously [26].

### Statistical analysis

GraphPad Prism 5 software (GraphPad Software Inc.) was used. Data represent the mean ± SD. Statistical analyses were performed using Student’s *t*-test, a one-way or two-way analysis of variance (ANOVA) followed by the Tukey–Kramer test for group comparisons. Survival curves were generated by the Kaplan–Meier method, and statistical comparisons were made using the log-rank method. The levels of statistical significance are shown as asterisks or letters and defined in each figure legend together with the name of the statistical test that was used. A *p* value of *P* < 0.05 was considered statistically significant.

## Results

### Cytotoxicity of piperaceae extracts and sulfadiazine

To analyze the toxicity of each piperaceae extract on HFF cells *in vitro*, we examined cell proliferation using a CCK-8 cell counting kit. When exposed to 100 μg/mL of *P*. *betle* extract for 24 h, the cell proliferation rate was 66.19%, while the proliferation rates of HHF cells treated with either 1, 5, 10, 25 or 50 μg/mL of *P*. *betle* extract were more than 100% (S2A Fig). Therefore, the safe concentration of *P*. *betle* extract was considered to be < 50 μg/mL in this study. However, the proliferation rates of the HFF cells treated with 100 μg/mL of *P*. *nigrum* or *P*. *sarmentosum* extract were more than 100% (S2A Fig), indicating that these extracts had lower cytotoxicity than that of the *P*. *betle* extract. The IC_50_ value of the *P*. *betle* extract against HFF cell growth was 180.2 μg/mL (S2B Fig). There was no obvious cytotoxic effect of sulfadiazine on the HFF cells, even at the highest concentration (1 mg/mL) (S3C Fig).

### Effects of the piperaceae extracts and sulfadiazine on *T*. *gondii* growth *in vitro*

**Fig 1:**
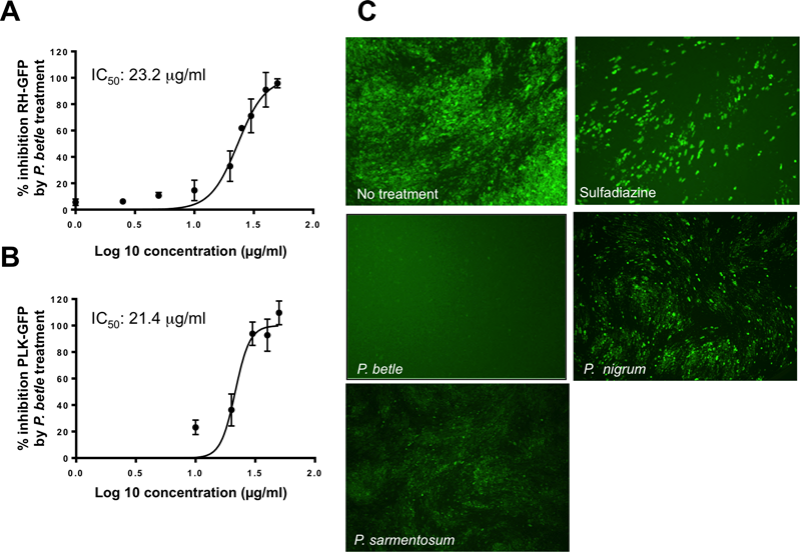
Anti-*Toxoplasma* activity of *P*. *betle* extract on RH-GFP and PLK-GFP Anti-*Toxoplasma* activity of the *P*. *betle* extract on intracellular parasites RH-GFP (A) and PLK-GFP (B). The RH-GFP and PLK-GFP-infected HFF cells were treated with the *P*. *betle* extract for 72 h at different concentrations from 0 to 50 μg/mL. Data represent the mean values ± SD for three independent experiments. The IC_50_ values of the *P*. *betle* extract on RH-GFP and PLK-GFP were 23.2 g/mL and 21.4 μg/mL, respectively. (C) Representative images of *T*. *gondii* RH-GFP-infected HFF cells treated with sulfadiazine (1 mg/mL), or either *P*. *betle* (50 μg/mL), *P*. *nigrum* (50 μg/mL) or *P*. *sarmentosum* (50 μg/mL) extract.

To analyze the anti-*Toxoplasma* effects of each piperaceae extract *in vitro*, we examined the fluorescence intensity of RH-GFP. At 72 h post-treatment, the *P*. *betle* extract inhibited RH-GFP growth at concentrations 25 and 50 μg/mL showing 63.4 ± 16.9% and 96.8 ± 7.2% inhibition, respectively (Fig 1A). However, the *P*. *nigrum* and *P*. *sarmentosum* extracts had no effects on RH-GFP growth (Fig 1C). The IC_50_ of the *P*. *betle* extract on RH-GFP was 23.2 μg/mL, while those of the *P*. *sarmentosum* and *P*. *nigrum* extracts were > 100 μg/mL. Futhermore, the *P*. *betle* extract also inhibited the growth of PLK-GFP (IC_50_: 21.4 μg/mL) (Fig 1B). Although sulfadiazine inhibited the growth of *T*. *gondii* (IC_50_ on RH-GFP: 99.4 μg/mL, IC_50_ on PLK-GFP: 22.3 μg/mL), the GFP signal was still observed, even at the highest concentration of sulfadiazine (1 mg/mL) (S3 Fig).

#### Effects of the piperaceae extracts on intracellular and extracellular *T*. *gondii in vitro*

**Fig 2:**
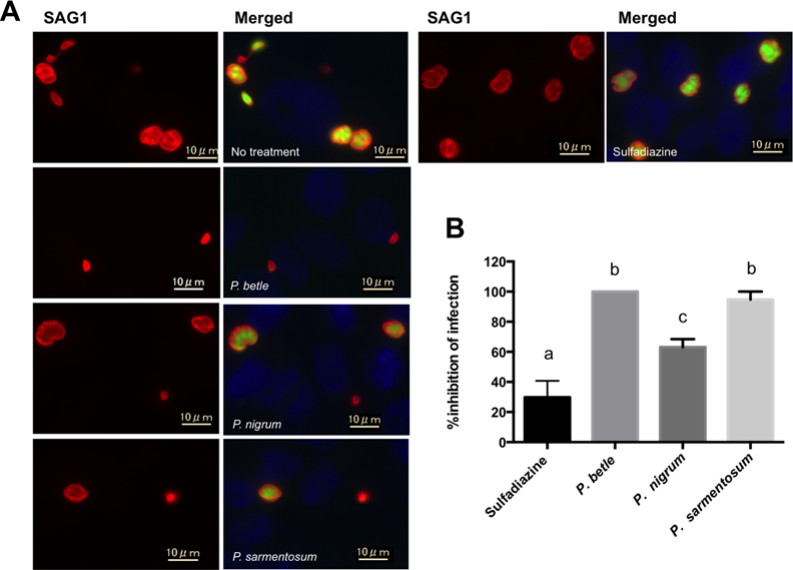
Effects of the three piperaceae extracts at 25 μg/mL and sulfadiazine at 1 mg/mL on extracellular *T*. *gondii*. The RH-GFP line, pre-treated with either of the extracts (*P*. *betle*, *P*. *nigrum* or *P*. *sarmentosum*) or sulfadiazine for 1 h, was then used to infect vero cells. After 24 h, the infected cells were analyzed by IFAT to measure the infection rates. (A) Representative images of *T*. *gondii* RH-GFP-infected vero cells. The cells were treated with either *P*. *betle* (25 μg/mL), *P*. *nigrum* (25 μg/mL), *P*. *sarmentosum* (25 μg/mL) extract or sulfadiazine (1 mg/mL). SAG1, red; GFP, green; nucleus, blue. (B) The % inhibition of infection for RH-GFP was measured by counting the number of SAG1-positive vero cells per 100 vero cells. Each bar represents the mean ± SD of three wells per group. The results represent two independent experiments. The different letters above the data bars in the graphs indicate statistically significant differences as determined by a one-way ANOVA plus Tukey–Kramer post-hoc analysis (*P* < 0.05).

To examine the effect of each piperaceae extract on extracellular *T*. *gondii*, purified extracellular parasites pretreated with 25 μg/mL of either extract were used to infect vero cells (Fig 2A). Pretreatment with the *P*. *betle* extract resulted in 100% inhibition of the parasite infection while pretreatment with extracts from *P*. *sarmentosum* or *P*. *nigrum* resulted in 94.7 ± 5.3% and 63.2 ± 5.3% inhibition, respectively (Fig 2B). Treatment with the *P*. *betle* extract resulted in no GFP signal from the RH-GFP parasites (Fig 2A), suggesting that destruction of the parasite cell membrane and release of GFP from the cytosol had occurred. All extracts were more effective at parasite inhibition than 1 mg/mL of sulfadiazine (29.8 ± 10.9%) (Fig 2B).

**Fig 3:**
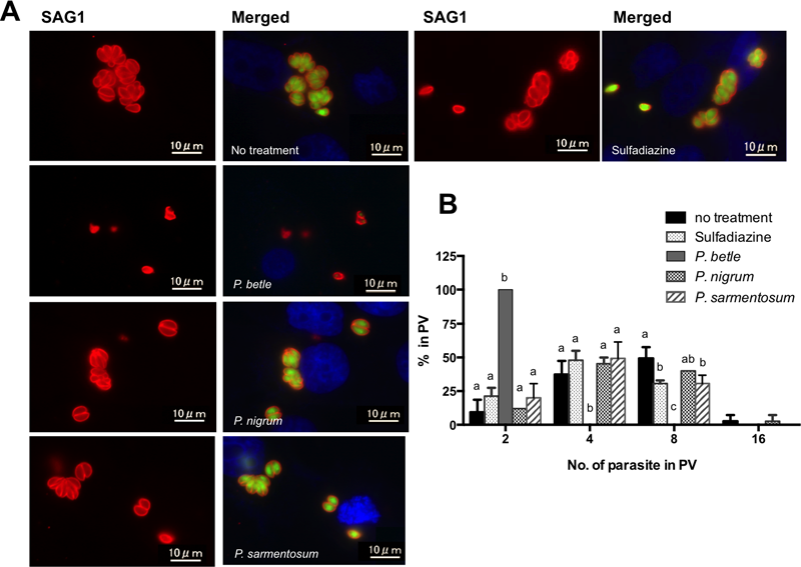
Effects of the three piperaceae extracts at 25 μg/mL and sulfadiazine at 1 mg/mL on intracellular *T*. *gondii*. The RH-GFP-infected vero cells were treated with either of the three piperaceae extracts or sulfadiazine for 72 h, and were then analyzed by IFAT to measure the parasitophorous vacuole (PV) sizes. (A) Representative images of *T*. *gondii* RH-GFP-infected vero cells. The cells were treated with either *P*. *betle* (25 μg/mL), *P*. *nigrum* (25 μg/mL), *P*. *sarmentosum* (25 μg/mL) extract, or sulfadiazine (1 mg/mL). SAG1, red; GFP, green; nucleus, blue. (B) The number of parasites in PVs was measured by counting the number of SAG1-positive parasites per PV. Each bar represents the mean ± SD of three wells per group. Results represent two independent experiments. The different letters above the data bars in the graphs indicate statistically significant differences in the number of parasites in PVs as determined by two-way ANOVA plus Tukey–Kramer post-hoc analysis (*P* < 0.05).

To test the effect of each piperaceae extract on intracellular *T*. *gondii*, RH-GFP-infected vero cells were treated with 25 μg/mL of either extract or 1 mg/mL of sulfadiazine (Fig 3A). Only two parasites per PV were found in the cells treated with the *P*. *betle* extract at 24 h post-infection (Fig 3B). Additionally, the GFP signal from the parasites was lower in cells treated with the *P*. *betle* extract (Fig 3A), indicating the anti-*Toxoplasma* effect of this extract. Sulfadiazine or *P*. *sarmentosum* treatment caused only slight inhibition of parasite replication (Fig 3B).

#### Effects of the piperaceae extracts on *T*. *gondii* infections *in vivo*

**Fig 4:**
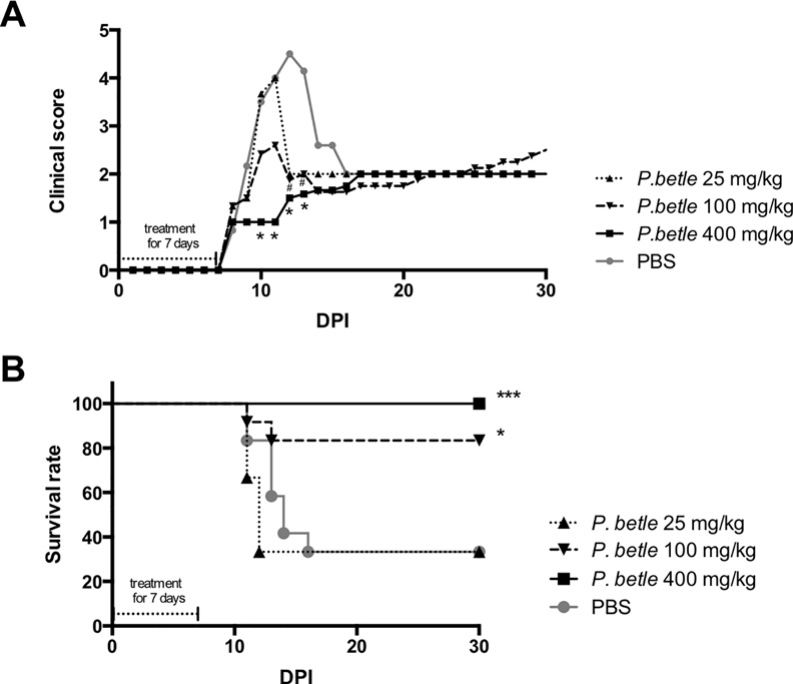
Clinical scores and survival of *T*. *gondii*-infected mice treated with *P*. *betle* extract. Mice were intraperitoneally administrated with *P*. *betle* extracts at 25, 100 and 400 mg/kg/day or PBS from 1 to 7 days post-infection with 10^3^ PLK tachyzoites per mouse. Clinical scores (A) and survival (B) were monitored for 30 days post-infection in the mice. The clinical scores represent the mean total values for all mice used in this study. Data represent the mean values of all the mice used in two independent experiments performed together (*P*. *betle* extract at 100 and 400 mg/kg/day, PBS, n = 6 + 6; *P*. *betle* extract at 25 mg/kg/day, n = 6). The clinical scores were analyzed by two-way ANOVA plus Tukey–Kramer post-hoc analysis at the time points indicated (*Difference between PBS and *P*. *betle* extract at 400 mg/kg/day, *P* < 0.05; # Difference between PBS and *P*. *betle* extract at 100 mg/kg/day, *P* < 0.05). Survival curves were generated with the Kaplan–Meier method. According to the log-rank test, the differences between the PBS and *P*. *betle* extracts were significant (*Difference between PBS and *P*. *betle* extract at 100 mg/kg/day, *P* < 0.05; *** Difference between PBS and *P*. *betle* extract at 400 mg/kg/day, *P* < 0.001).

Because the *P*. *betle* extract had anti-*T*. *gondii* activity *in vitro*, we evaluated the effects of the *P*. *betle* extract on *T*. *gondii in vivo* (Fig 4). Although higher clinical scores were seen in the PBS-injected mice from 10 to 13 days post infection (dpi), treatment with *P*. *betle* extract at 400 and 100 mg/kg reduced the clinical signs from 10 to 13 dpi and from 12 to 13 dpi, respectively (Fig 4A). Furthermore, treating the infected mice with 400 and 100 mg/kg of the *P*. *betle* extract resulted in 100% and 83.3% mouse survival, respectively (Fig 4B). However, the survival rates of the mice treated with 25 mg/kg of *P*. *betle* extract or PBS was 33.3% (Fig 4B). This result indicates that treatment with 400 and 100 mg/kg of *P*. *betle* extract ameliorated toxoplasmosis in mice during the acute infection.

**Fig 5:**
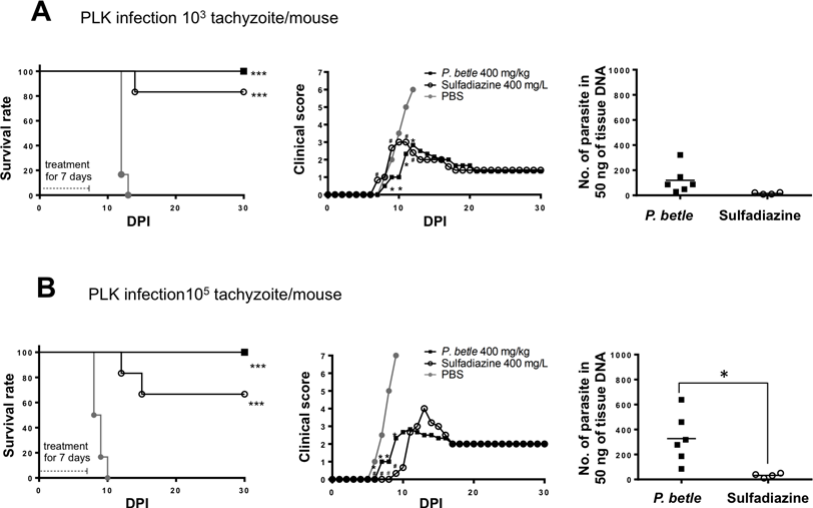
Survival, clinical score and parasite burden of mice infected with 1,000 and 100,000 *T*. *gondii* tachyzoites under treatment with *P*. *betle* extract or sulfadiazine. Mice infected with 1,000 (A) and 100,000 tachyzoites (B) of the *T*. *gondii* PLK strain (n = 6) were treated with 400 mg/kg *P*. *betle* extract, PBS via the intraperitoneal route, or 400 mg/L sulfadiazine via drinking water as a standard treatment from 1 to 7 days post-infection (dpi). Survival and clinical scores were monitored for 30 dpi and the brains from the surviving mice were collected to determine the parasite burden at 30 dpi. Survival curves were generated by the Kaplan–Meier method. According to the log-rank test, the differences between the PBS and *P*. *betle* extracts were significant (***Difference between PBS and *P*. *betle* extract or sulfadiazine treatment, *P* < 0.001). The clinical scores represent the mean total values for all the mice used in this study. Data represent the mean values of all the mice. The clinical scores were analyzed by two-way ANOVA plus Tukey–Kramer post-hoc analysis at the time points indicated (*Difference between PBS and *P*. *betle* extract, *P* < 0.05; # Difference between PBS and sulfadiazine, *P* < 0.05). The number of parasites in 50 ng of tissue DNA per individual (symbols) and the mean levels (horizontal lines) are indicated. A significant difference between the two groups was observed by a student’s *t*-test. (*Difference between treatment with sulfadiazine and *P*. *betle* extract, *P* < 0.05)

To evaluate further the anti-*Toxoplasma* activity of *P*. *betle* extract, we performed additional experiments to compare the extract-treated group of mice with the sulfadiazine-treated mouse group (Fig 5). In the case of infection with 1,000 tachyzoites, the survival rate of the *P*. *betle*–treated mouse group was 100%, while one mouse died at 14 dpi in sulfadiazine-treated group and all mice died within 13 dpi in the PBS-injected group (Fig 5A). The clinical scores of the *P*. *betle*- and sulfadiazine-treated mice against infection with 1,000 *T*. *gondii* were significantly lower than those of the PBS-injected mice from 9 to 12 dpi and 11 to 12 dpi, respectively (Fig 5A). There was no significant difference in the parasite numbers in the brains of the surviving mice between the *P*. *betle*–treated and sulfadiazine-treated groups (Fig 5A). Furthermore, all mice in the *P*. *betle*–treated group survived the infection with 100,000 tachyzoites, but two mice died at 12 and 15 dpi in the sulfadiazine-treated group, and all mice died within 10 dpi in the PBS-injected group (Fig 5B). Treatment of the infected mice with *P*. *betle* or sulfadiazine decreased the clinical signs during the acute phase of the infection from 6 to 9 dpi (Fig 5B). The clinical score from 7 to 9 dpi and the number of parasites in the brains of the surviving mice in the sulfadiazine-treated group were significantly lower than those of *P*. *betle*–treated group (Fig 5B). Altogether, treatment with the *P*. *betle* extract controlled acute toxoplasmosis in the mice, although some parasites were detectable in their brains.

## Discussion

Toxoplasmosis is one of the most important and challenging diseases in public health. To control *T*. *gondii* infection and the toxoplasmosis caused by it, herbal medicine and natural herb extracts are of growing interest. There are many traditional herbal medicines with antimicrobial and antihelminthic properties, and some have anti-*Toxoplasma* activities such as *Curcuma longa* [27], *Eurycoma longifolia* Jack [28,29], and *Myristica fragrans* Houtt [30]. Moreover, the anti-*T*. *gondii* activity of *Artemisia annua* L. [5,31,32], *Dichroa febrifuga* [33], herbal extracts from South Korea (*Sophora flavescens*, *Sinomenium acutum*, *Pulsatilla koreana*, *Ulmus macrocarpa* and *Torilis japonica*) [34], and *Eurycoma longifolia* [29] have been reported. However, piperaceae extracts have not been tested for their potential anti-*Toxoplasma* effects even though they have many pharmacological properties including activities against fungi [35], insects [8], protozoa [11,17,36], helminthes [37,38] and cancer cells [12]. Only extracts of *P*. *nigrum* have been shown to possess *in vivo* activity, with a reported *T*. *gondii* growth inhibition of 78.3% and 86.3% with treatment doses 100 and 200 mg/kg/day, respectively [27], but there is no *in vitro* information for this extract. Herein, we evaluated the effects of ethanol extracts of *P*. *betle*, *P*. *nigrum* and *P*. *sarmentosum* from Thailand on *T*. *gondii* growth *in vitro* and *in vivo*.

Importantly, the *P*. *betle* extract had anti-*Toxoplasma* activity both *in vitro* and *in vivo*. In the *in vitro* tests, 25 μg/mL of the *P*. *betle* extract eradicated extracellular and intracellular parasites. The selective activity of the treatment was considered through the selectivity index (SI). Both *P*. *betle* extract and sulfadiazine were calculated as the ratio between cytotoxic IC_50_ values and parasitic IC_50_ values [39]. The IC_50_ values of the *P*. *betle* extract for HFF cells, RH-GFP and PLK-GFP were 180.2 μg/mL, 23.2 μg/mL and 21.4 μg/mL, respectively. Therefore, each SI for RH-GFP and PLK-GFP was 7.77 and 8.42, respectively. Because there was no obvious cytotoxic effect of sulfadiazine on the HFF cells, even at the highest concentration (1 mg/mL), the SI was not able to be used in our study. In a previous study, de Oliveira et al [5] reported that the IC_50_ value of sulfadiazine on *T*. *gondii* (RH strain) in HFF cells was 70 μg/mL, and the viability of HFF cells in the presence of 200 μg/mL of sulfadiazine decreased by 28% (no SI value was reported). Schoondermark-van de Ven et al [40] reported that the IC_50_ of sulfadiazine against the growth of *T*. *gondii* in human epithelial type 2 (HEp-2) cells could not be determined because the drug was toxic to HEp-2 cells at concentrations above 1000 μg/mL. Thus, in comparision with sulfadiazine, *P*. *betle* extract should be effective at controlling the growth of *T*. *gondii in vitro*.

Furthermore, treatment of *T*. *gondii*-infected mice with the *P*. *betle* extract increased the survival rates of the mice, particularly at the highest concentration (400 mg/kg) of extract that we used. When compared with sulfadiazine treatment, treatment with *P*. *betle* extract (400 mg/kg) produced better mouse survival rates, although *T*. *gondii* DNA was still detectable in the mouse brains. Thus, treatment with *P*. *betle* extract was effective at controling acute toxoplasmosis in mice. In this study, we did not test the toxicity of *P*. *betle* extract *in vivo*, but a previous study showed that an ethanol extract of *P*. *betle* leaves (after oral administration of 1,500 mg/kg/day for 40 consecutive days) produced no significant side-effects to cross-bred male albino rats in terms of clinical signs, food intake, percent weight gain and serum parameters [41], indicating that our regimen should be reliable.

Extracts of *P*. *nigrum* and *P*. *sarmentosum* were also tested *in vitro* in our study. *P*. *nigrum* and *P*. *sarmentosum* each showed an inhibitory effect against extracellular *T*. *gondii*, but not against intracellular parasites. Our results indicate that *P*. *nigrum* and *P*. *sarmentosum* extracts affected the extracellular parasites, while the *P*. *betle* extract was effective against extracellular and intracellular parasites alike. The main chemical classes found in *P*. *betle* leaves are alkaloids, terpenes, anthraquinones, flavonoids, tannins, saponins and steroids [17]; more specifically, chavibetol, chavibetol acetate, allylpyrocatechol diacetate, eugenol, safrole, quercetin, a-Pinene, f-pinene, u-limonene, and saprobe [14,17,42,43]. Furthermore, phytochemical compounds such as alkaloids and flavonoids have antiplasmodial activities [17]. Therefore, any one or a combination of these compounds might affect *T*. *gondii* growth. In a future study, we will test the effects of the phytochemical compounds that were found in *P*. *betle* extract for their anti-*Toxoplasma* activities.

## Conclusion

In the present study, we found that *P*. *betle* had little toxicity to HFF cells and effectively inhibited *T*. *gondii in vitro* and *in vivo*. These results indicate that *P*. *betle* extract contains active ingredients with potential for treating toxoplasmosis.

## Supporting Information

S1 Fig Plant materials. (A) *Piper betle* L., (B) *P*. *nigrum* L., and (C) *P*. *sarmentosum* Roxb. were identified and transferred to the herbarium by the Faculty of Pharmacy, Mahidol University, 447 Sri-Ayuthaya Road. Rajathevi Bankok 10400, Thailand. The serial numbers given were as follows: *P*. *betle* L.: PBM05160, *P*. *nigrum* L.: PBM05159, *P*. *sarmentosum* Roxb.: PBM05161.
(PDF)

S2 Fig Cyotoxicity of the piperaceae extracts. (A) Cytotoxicity testing of HFF cells after treatment with either one of three piperaceae extracts (*P*. *betle*, *P*. *nigrum*, or *P*. *sarmentosum*) at concentrations of 0 to 100 μg/mL for 24 h. Data represent the mean values ± SD for three independent experiments. (B) Inhibition HFF cell growth by the *P*. *betle* extract. HFF cells were exposed to *P*. *betle* extract at concentrations 0 to 500 μg/mL. Data represent the mean values ± SD for three independent experiments. The IC_50_ value of the *P*. *betle* extract on HFF cells was calculated based on three independent experiments performed together.
(PDF)

S3 Fig Anti-*Toxoplasma* activity of sulfadiazine on RH-GFP and PLK-GFP, and the cyotoxicity of sulfadiazine to HFF cells. Anti-*Toxoplasma* activity of sulfadiazine against RH-GFP (A) and PLK-GFP parasites (B). The RH-GFP and PLK-GFP-infected HFF cells were treated with sulfadiazine for 72 h at concentrations of 10 ng/mL to 1 mg/mL and the IC_50_ values were calculated for RH-GFP and PLK-GFP. Data represent the mean values ± SD for three independent experiments. (C) Inhibition of HFF cell growth by sulfadiazine. HFF cells were exposed to sulfadiazine at the highest concentration of 1 mg/mL. Data represent the mean values ± SD (n = 3). (D) Representative images of *T*. *gondii* RH-GFP- and PLK GFP-infected HFF cells treated with sulfadiazine (1 mg/mL) and culture medium alone (no treatment).
(PDF)
