# Supplementary material for: Effects of Extracts from Thai Piperaceae Plants against Infection with Toxoplasma gondii
Source: PLoS One. 2016 May 23;11(5):e0156116. doi: 10.1371/journal.pone.0156116 (PMC4877092; doi:10.1371/journal.pone.0156116)
Supplement: S1 Fig — (A) Piper betle L., (B) P. nigrum L., and (C) P. sarmentosum Roxb. were identified and transferred to the herbarium by the Faculty of Pharmacy, Mahidol University, 447 Sri-Ayuthaya Road. Rajathevi Bankok 10400, Thailand. The serial numbers given were as follows: P. betle L.: PBM05160, P. nigrum L.: PBM05159, P. sarmentosum Roxb.: PBM05161. (PDF) [file pone.0156116.s001.pdf]

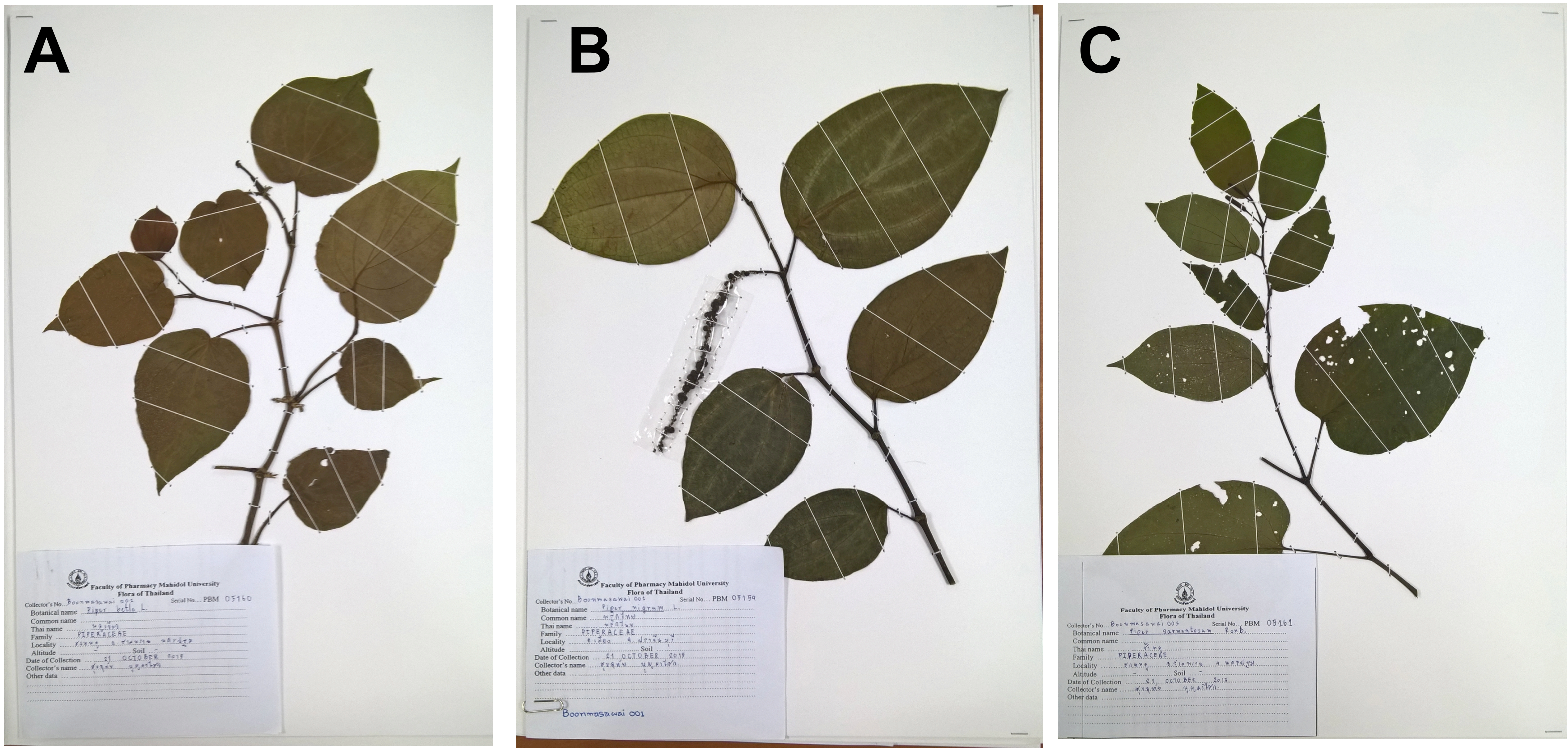

S1 Fig. Plant materials (A) *Piper betle* L., (B) *Piper nigrum* L., and (C) *Piper sarmentosum* Roxb. were identified and transferred to the herbarium by the Faculty of Pharmacy, Mahidol University, 447 Sri-Ayuthaya Road. Rajathevi Bankok 10400, Thailand. The serial numbers given were as follows: *P. betle* L.: PBM05160, *P. nigrum* L.: PBM05159, *P. sarmentosum* Roxb.: PBM05161.
