# Supplementary material for: Effects of Extracts from Thai Piperaceae Plants against Infection with Toxoplasma gondii
Source: PLoS One. 2016 May 23;11(5):e0156116. doi: 10.1371/journal.pone.0156116 (PMC4877092; doi:10.1371/journal.pone.0156116)
Supplement: S2 Fig — (A) Cytotoxicity testing of HFF cells after treatment with either one of three piperaceae extracts (P. betle, P. nigrum, or P. sarmentosum) at concentrations of 0 to 100 μg/mL for 24 h. Data represent the mean values ± SD for three independent experiments. (B) Inhibition HFF cell growth by the P. betle extract. HFF cells were exposed to P. betle extract at concentrations 0 to 500 μg/mL. Data represent the mean values ± SD for three independent experiments. The IC50 value of the P. betle extract on HFF cells was calculated based on three independent experiments performed together. (PDF) [file pone.0156116.s002.pdf]

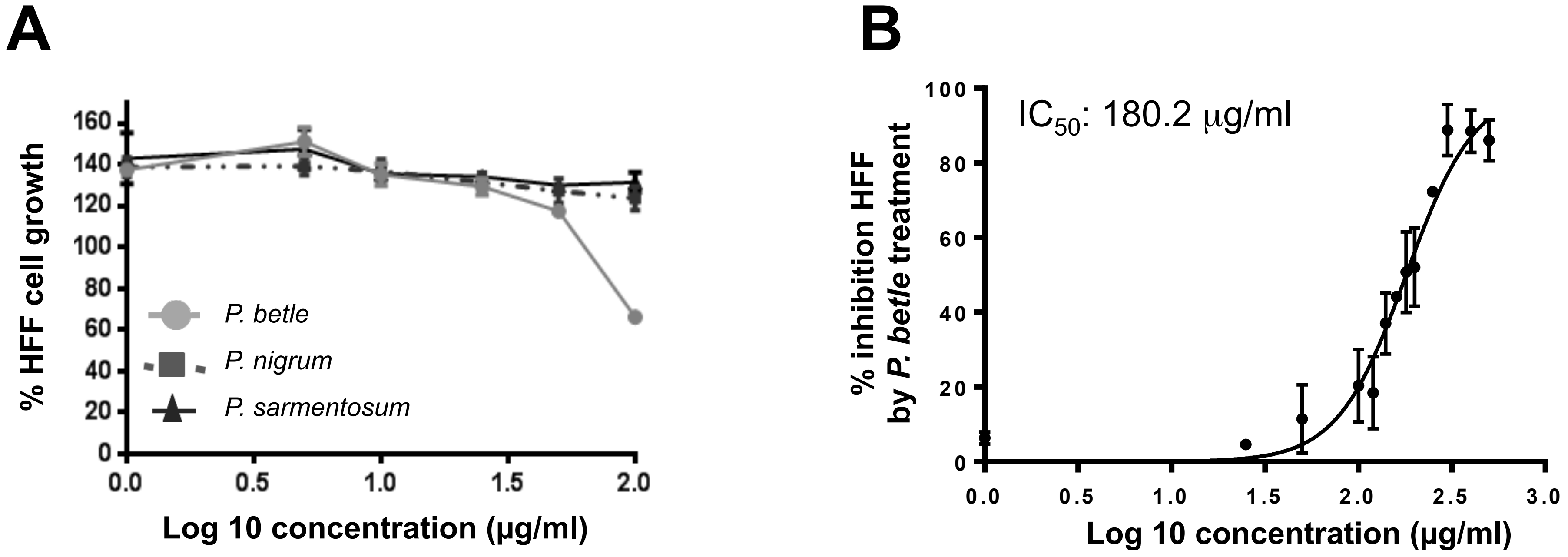

S2 Fig. Cytotoxicity of the piperaceae extracts (A) Cytotoxicity testing of HFF cells after treatment with either one of three piperaceae extracts (*P. betle*, *P. nigrum*, or *P. sarmentosum*) at concentrations of 0 to 100 µg/mL for 24 h. Data represent the mean values ± SD for three independent experiments. (B) Inhibition HFF cell growth by the *P. betle* extract. HFF cells were exposed to *P. betle* extract at concentrations 0 to 500 µg/mL. Data represent the mean values ± SD for three independent experiments. The IC_{50} value of the *P. betle* extract on HFF cells was calculated based on three independent experiments performed together.
