# Supplementary material for: Effects of Extracts from Thai Piperaceae Plants against Infection with Toxoplasma gondii
Source: PLoS One. 2016 May 23;11(5):e0156116. doi: 10.1371/journal.pone.0156116 (PMC4877092; doi:10.1371/journal.pone.0156116)
Supplement: S3 Fig — Anti-Toxoplasma activity of sulfadiazine against RH-GFP (A) and PLK-GFP parasites (B). The RH-GFP and PLK-GFP-infected HFF cells were treated with sulfadiazine for 72 h at concentrations of 10 ng/mL to 1 mg/mL and the IC50 values were calculated for RH-GFP and PLK-GFP. Data represent the mean values ± SD for three independent experiments. (C) Inhibition of HFF cell growth by sulfadiazine. HFF cells were exposed to sulfadiazine at the highest concentration of 1 mg/mL. Data represent the mean values ± SD (n = 3). (D) Representative images of T. gondii RH-GFP- and PLK GFP-infected HFF cells treated with sulfadiazine (1 mg/mL) and culture medium alone (no treatment). (PDF) [file pone.0156116.s003.pdf]

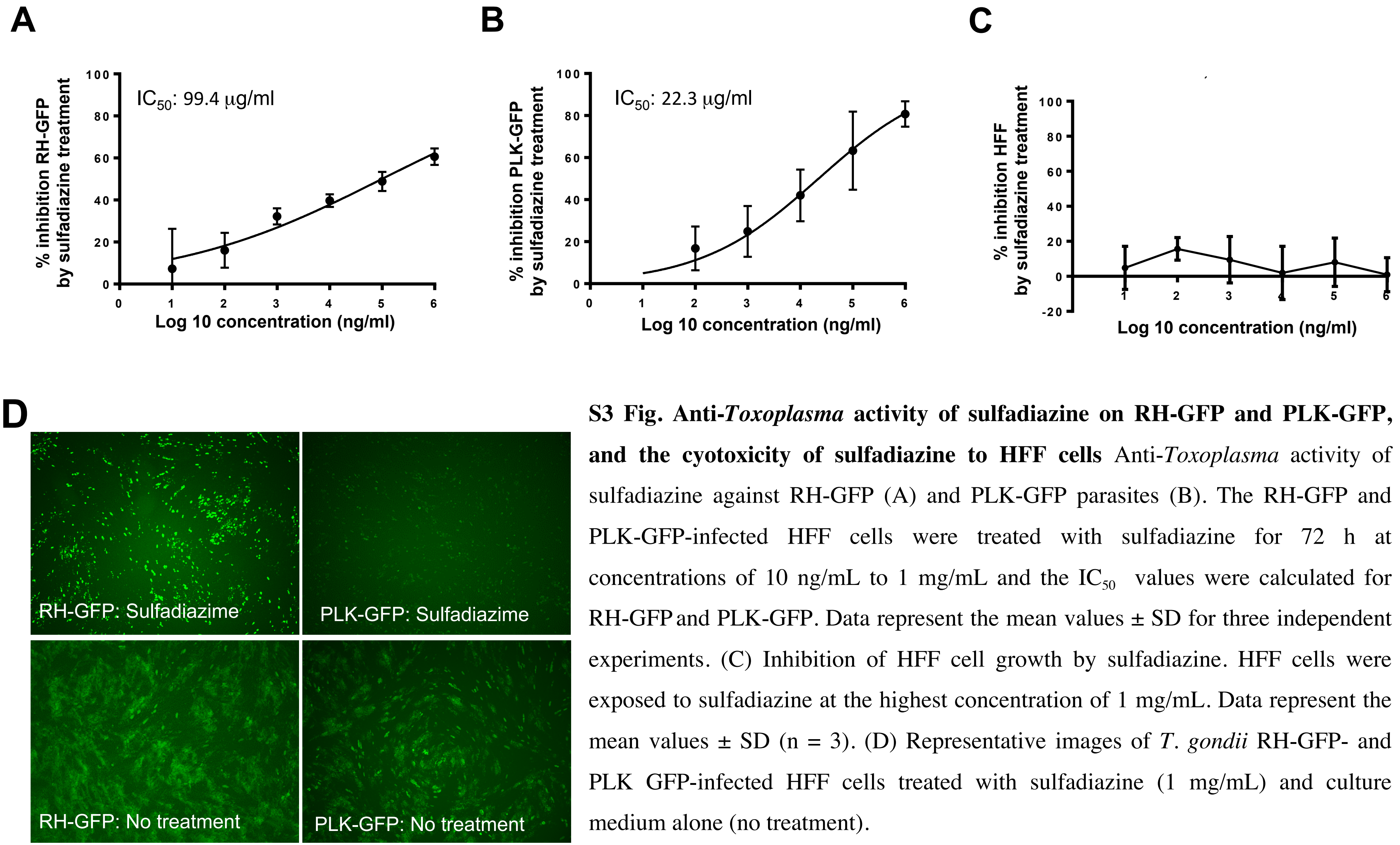


**S3 Fig. Anti-*Toxoplasma* activity of sulfadiazine on RH-GFP and PLK-GFP, and the cytotoxicity of sulfadiazine to HFF cells** Anti-*Toxoplasma* activity of sulfadiazine against RH-GFP (A) and PLK-GFP parasites (B). The RH-GFP and PLK-GFP-infected HFF cells were treated with sulfadiazine for 72 h at concentrations of 10 ng/mL to 1 mg/mL and the $IC_{50}$ values were calculated for RH-GFP and PLK-GFP. Data represent the mean values ± SD for three independent experiments. (C) Inhibition of HFF cell growth by sulfadiazine. HFF cells were exposed to sulfadiazine at the highest concentration of 1 mg/mL. Data represent the mean values ± SD (n = 3). (D) Representative images of *T. gondii* RH-GFP- and PLK GFP-infected HFF cells treated with sulfadiazine (1 mg/mL) and culture medium alone (no treatment).
